# An Approach to Early Detection of Metabolic Syndrome through Non-Invasive Methods in Obese Children

**DOI:** 10.3390/children7120304

**Published:** 2020-12-17

**Authors:** Rafael Molina-Luque, Natalia Ulloa, Andrea Gleisner, Martin Zilic, Manuel Romero-Saldaña, Guillermo Molina-Recio

**Affiliations:** 1 Grupo Asociado de Investigación Estilos de Vida, Innovación y Salud, Instituto Maimónides de Investigación Biomédica de Córdoba (IMIBIC), 14004 Córdoba, Spain; rafael.moluq@gmail.com (R.M.-L.); manuelromerosal@gmail.com (M.R.-S.); en1moreg@uco.es (G.M.-R.); 2 Departamento de Enfermería, Farmacología y Fisioterapia, Facultad de Medicina y Enfermería, Universidad de Córdoba, 14004 Córdoba, Spain; 3 Centro de Vida Saludable y Departamento de Bioquímica Clínica e Inmunología, Facultad de Farmacia, Universidad de Concepción, Concepción 4070386, Chile; 4 Departamento de Pediatría, Facultad de Medicina, Universidad de Concepción, Concepción 4070386, Chile; andrea.gleisner@gmail.com; 5 Facultad de Medicina, Universidad de Concepción, Concepción 4070386, Chile; mzilic@udec.cl

**Keywords:** anthropometry, child, early diagnosis, metabolic syndrome, obesity

## Abstract

Background: Metabolic Syndrome (MetS) has a high prevalence in children, and its presence increases in those with a high BMI. This fact confirms the need for early detection to avoid the development of other comorbidities. Non-invasive variables are presented as a cost-effective and easy to apply alternative in any clinical setting. Aim: To propose a non-invasive method for the early diagnosis of MetS in overweight and obese Chilean children. Methods: We conducted a cross-sectional study on 221 children aged 6 to 11 years. We carried out multivariate logistic regressions, receiver operating characteristic curves, and discriminant analysis to determine the predictive capacity of non-invasive variables. The proposed new method for early detection of MetS is based on clinical decision trees. Results: The prevalence of MetS was 26.7%. The area under the curve for the BMI and waist circumference was 0.827 and 0.808, respectively. Two decision trees were calculated: the first included blood pressure (≥104.5/69 mmHg), BMI (≥23.5 Kg/m^2^) and WHtR (≥0.55); the second used BMI (≥23.5 Kg/m^2^) and WHtR (≥0.55), with validity index of 74.7% and 80.5%, respectively. Conclusions: Early detection of MetS is possible through non-invasive methods in overweight and obese children. Two models (Clinical decision trees) based on anthropometric (non-invasive) variables with acceptable validity indexes have been presented. Clinical decision trees can be applied in different clinical and non-clinical settings, adapting to the tools available, being an economical and easy to measurement option. These methods reduce the use of blood tests to those patients who require confirmation.

## 1. Introduction

Metabolic Syndrome (MetS) is defined as a group of cardiometabolic disorders that increase the risk of suffering from cardiovascular diseases and type 2 diabetes, pathologies that are among the leading causes of death worldwide [1,2]. Abdominal obesity, low levels of high-density lipoprotein cholesterol (Chol-HDL), and elevated blood pressure, triglycerides, and fasting blood glucose are essential alterations [3].

MetS prevalence has increased in the last years and has become a public health problem due to the increase in morbi-mortality [4,5,6]. Although it has generally been considered a typical affectation of the adult population, it increasingly appears in a higher proportion in the younger population groups [7]. This fact has been related to the exponential increase in the prevalence of overweight and obesity. These conditions are linked to insulin resistance, one of the physiopathological mechanisms of MetS [8,9]. Prevalence among children ranges from 3.3 to 29.2%, with higher values among children with higher BMI [10]. The Chilean population is not an exception to this problem. The latest national health survey revealed that the prevalence of MetS is around 40.1% in the adult population [11]. Several studies show oscillations between 20% and 40% in the child population, depending on the selected diagnostic criteria, regions where the study was carried out, and overweight and obesity prevalence in those areas [12,13].

This high proportion of MetS has led to looking for methods to detect it early, limiting the development of associated comorbidities. Among the tools proposed for early diagnosis, those that do not require invasive techniques stand out. These guarantee their use in diverse clinical settings, especially in places with scarce healthcare resources, and offer a high cost-efficiency, as they do not require blood tests [14,15]. In this sense, several works have shown that the waist to height ratio (WHtR) has a high predictive capacity in the adult population. Besides, they have provided methods with high validity indexes together with other parameters such as blood pressure [16]. On this point, several authors have denoted that the anthropometric variables are the most appropriate for early detection of MetS in children. Neck circumference, waist circumference, or BMI are associated with MetS at pediatric ages [17,18]. However, although the variables are linked separately with MetS, no work has proposed a non-invasive algorithm to improve children’s screening at high risk for this syndrome.

Therefore, this study aims to propose a method that uses non-invasive variables for the early detection of MetS in overweight and obese Chilean children.

## 2. Materials and Methods

### 2.1. Design, Population, Sample

A cross-sectional study was carried out on boys and girls between the ages of 6 and 11 in the urban area of the city of Hualpén in the Biobío Region, Chile in 2010. The minimum sample size was 188 for an expected prevalence of 22.7% [12] in a population of 1,550,248 [19], 6% precision and a 95% confidence interval (CI). The final sample consisted of 221 boys and girls.

We included children whose parents signed informed consent. We excluded those suffering from some chronic pathology.

### 2.2. Study Variables and Measurements

The diagnosis of Metabolic Syndrome was determined following the criteria established by Cook [20], defined by the presence of three or more of the following alteration: waist circumference (WC) ≥ 90th percentile, systolic blood pressure (SBP) or diastolic (DBP) ≥ 90th percentile, HDL ≥ 40 mg/dL, triglycerides ≥ 110 mg/dL, and fasting glycemia levels ≥100 mg/dL, as established by the American Diabetes Association (ADA) [21].

The independent variables included were sex (boy, girl), age (years), and those grouped into:

- Anthropometric variables: weight (Kg), height (cm), BMI (Kg/m^2^), waist circumference (WC, cm), WHtR, fat-free mass (Kg), fat mass (Kg), fat mass percentage, SBP (mmHg) and DBP (mmHg).
- Analytical variables: glucose (mg/dL), Chol-HDL (mg/dL), Chol-LDL (mg/dL), triglycerides (mg/dL) and total cholesterol (mg/dL)

The anthropometric measurements were measured following the recommendations in the reference manual for anthropometric standardization [22]. Height was measured with a SECA 209 stadiometer with a precision of 0.1 cm. Weight and body composition (percentage of fat, fat mass, and fat-free mass) were measured with a bioimpedance meter (TANITA TBF-300, TANITA, Tokyo, Japan) with a precision of 0.1 kg. WC was measured halfway between the lower costal border and the iliac crest at the end of a normal aspiration, using a non-elastic flexible tape. The established recommendations were followed for measuring blood pressure (BP) [23], employing a calibrated digital sphygmomanometer (OMRON M3, OMRON, Kyoto, Japan). Analysis of circulating metabolites was performed from a fasting venous blood sample taken between 8.00 and 10.00 a.m. Measurements of lipid and glycidic indicators were performed using commercial kits (Cobas C11Roche, Indianapolis, IN, USA). Experienced technicians performed all the measurements to minimize the coefficient of variation. Each measure was made three times, and the average value was calculated.

The classification of nutritional status was performed following the criteria established by the World Health Organization for the BMI Z-Score [24]:

- Normal weight: Z-Score BMI > −2SD (standard deviation) and < +1SD;
- Overweight: Z-Score BMI > +1SD and < +2SD;
- Obesity: Z-Score BMI > +2SD.

To determine the presence of abdominal obesity (waist circumference > 90th percentile) and high blood pressure (HBP; SBP or DPB > 90th percentile), the reference tables were used according to age and sex, and height in the case of arterial pressure [25,26].

### 2.3. Ethical and Legal Aspects

The study was carried out in compliance with the fundamental principles laid down in the Declaration of Helsinki (1964), the Council of Europe Convention on Human Rights and Biomedicine (1997), the UNESCO Universal Declaration on the Human Genome and Human Rights (1997), as well as with the requirements laid down in Chilean legislation for biomedical research, the protection of personal data and bioethics. The Bioethics Committee of the Vice-Rectory of Research of the University of Concepción approved the study protocol (352-2019).

### 2.4. Statistical Analysis

The quantitative variables are presented with mean and standard deviation, and the qualitative values are shown with frequencies and percentages.

To assess the goodness of fit to a normal distribution of data from quantitative variables, the Kolmogorov-Smirnov test with the Lilliefors correction was used. To bivariate hypothesis contrast, the Student *t*-test for two means and the Mann–Whitney U was performed (using the Levene test for the contrast of homoscedasticity).

The multivariate analysis was performed through two statistical approaches:

- *Binary logistic regression models adjusted by diverse qualitative and quantitative predictive variables*. The Odds Ratio (OR) adjusted with its 95% confidence interval was determined. The goodness of fit tests (–2 log-likelihood, goodness of fit statistic, Cox and Snell R^2^, Nagelkerke R^2^ y Hosmer–Lemeshow tests) were calculated to evaluate the overall fit of the model.
- *Discriminant Analysis models adjusted only by quantitative predictive variables*. The coefficients for each of Fisher’s linear discriminant functions (MetS negative and MetS positive) were obtained. The Box M test was used to contrast the equalness of the matrixes for the two groups (MetS negative and MetS positive), and the Wilks Lambda test for contrasting the discriminant capacity compared with the predictive variables.

We also performed Receiver Operating Characteristic (ROC) curves and area under the curve (AUC) to determine each variable’s diagnostic accuracy to establish which best predicted the Metabolic Syndrome. Sensitivity, specificity, Youden, and validity indices were analyzed to determine its best cut off value for higher diagnostic accuracy.

The new MetS early detection model was obtained from a clinical decision tree (classification) using the chi-squared automatic interaction detection (CHAID) technique as a growth method. The statistical significance level for splitting nodes and merging categories was *p* < 0.05, significance values were corrected by the Bonferroni method, and the maximum number of iterations was 100. Various decision trees were produced with different modifications in the growth criteria, with a final minimum number of subjects of 50 and 30 in parent and child nodes, respectively.

The probability of an α error of below 5% (*p* < 0.05) was considered statistically significant for all the statistical analyses. The confidence interval was calculated at 95%. For the statistical analysis, IBM SPSS Statistics 22.0 software (IBM, Chicago, IL, USA) and Epidat 4.2. (Department of Sanidade, Xunta de Galicia, Galicia, Spain) were used.

## 3. Results

### 3.1. Prevalence of MetS and Anthropometric Predictor Variables

Of 221 children, 51.2% (95% CI 43.4–57%) were girls. The overall mean age was 9.1 ± 1.3 years (95% CI 8.9–9.3 years), with no significant differences between girls and boys (*p* > 0.05). Following the Z-Score BMI criteria, 67% (95% CI 60.3–73.1%) were overweight or obese, reaching the prevalence of 68.5% (95% CI 59–77%) and 65.5% (95% CI 55.8–74.3%) in girls and boys (*p* > 0.05), respectively. The prevalence of MetS observed in the sample was 26.7% (95% CI 21–33%), with a significantly higher proportion in girls than boys (OR = 2.2; *p* <0.05).

Table 1 shows the characteristics of the sample, the results of the independent variables for the groups with and without MetS and the analysis of raw and adjusted logistic regression. Of the variables studied, being overweight or obese (OR = 22.236; 95% CI 5.249–94.206; *p* < 0.001) and showing WHtR higher than or equal to 0.55 (OR = 16.763; 95% CI 5.801–48.441; *p* < 0.001), produced the greatest increase in the prevalence of MetS. We only took into account variables that did not require invasive procedures for the adjusted model. After adjusting for school children’s age and sex, only BMI and systolic blood pressure showed an association. 

**Figure 1:**
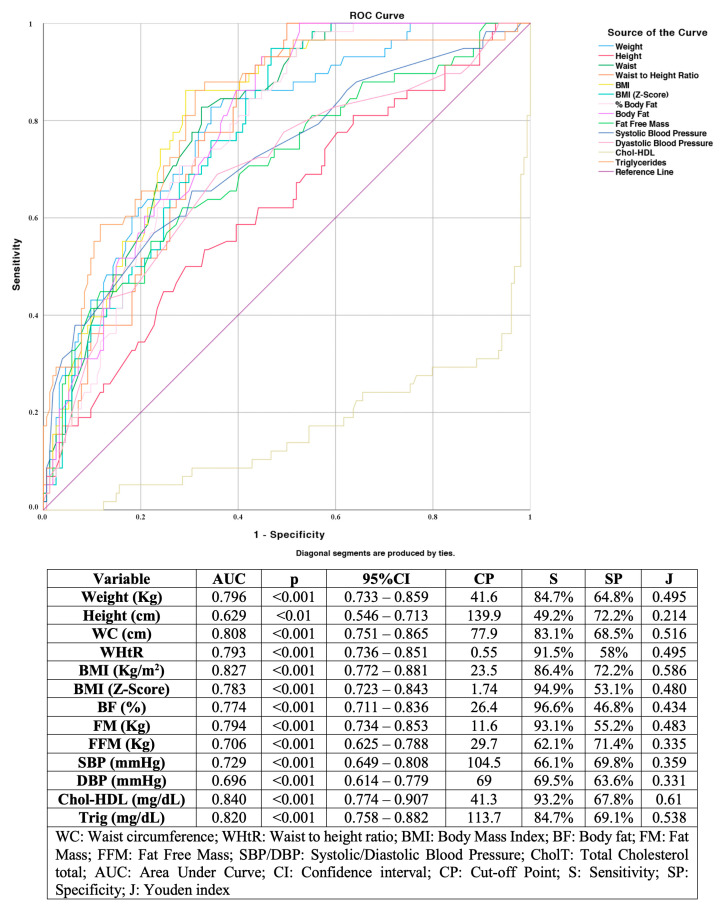
Receiver Operating Characteristic curves for independent variables.

Figure 1 shows the ROC curves, cut-off points, sensitivity, specificity, and Youden’s index of the variables that presented a significant MetS discrimination capacity. Chol-HDL showed an AUC of 84% and, for a cut-off point of 41.3 mg/dL, sensitivity and specificity of 93.2% and 67.8%, respectively. BMI obtained an AUC of 82.7% and, with a cut-off point of 23.5 kg/m^2^, 86.4% sensitivity, and 72.2% specificity.

### 3.2. Designing the Decision Tree to Detect MetS Based on Anthropometric Variables (Non-Invasive Method)

Two predictive models are proposed and compared based on the non-invasive variables that were statistically significant in the raw and adjusted logistic regression analysis.

- **Model 1:** based on sex (male and female) and dichotomization (according to whether the value is higher or lower than the cut-off point shown in Figure 1) of the WHtR (0.55), blood pressure (104.5/69 mmHg) and BMI (23.5 Kg/m^2^).
- **Model 2:** including sex (male and female), WC, HBP according to age, sex and height, and BMI (≥23.5 Kg/m^2^).

Table 2 shows the indicators’ results (sensitivity, specificity, and validity index) of each model. Model 1 and 2 have shown a sensitivity of 45.8% and 66.1%, and a specificity of 95.1% and 90.7%, respectively. As a complement to the models obtained by binary logistic regression, another was calculated through discriminant analysis. This model reached a sensitivity of 81.4%, a specificity of 75.3%, and a validity index of 76.9%. Table 3 shows these results and the coefficients for discriminant function variables.

**Figure 2:**
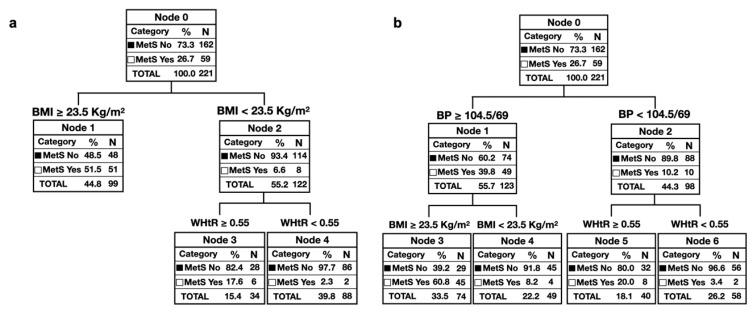
Clinical decision trees based on: (**a**) body mass index and weight to height ratio; (**b**) blood pressure, body mass index and weight to height ratio.

Finally, to offer a tool that is easy to understand and apply in clinical settings, several clinical decision trees were designed for the early detection of MetS. For this purpose, we used the variables shown to be significant in models 1 and 2. Figure 2 presents the decision trees (classification algorithm) with the best predictive capacity. The decision tree, shown in Figure 2a, reached a sensitivity of 86.4%, a specificity of 70.4%, and a validity index of 74.7%, being the BMI the variable with the highest association with the MetS ( χ^2^ = 56.446). The decision tree represented in Figure 2b performed a sensitivity, specificity, and validity index of 76.3%, 82.1%, and 80.5%, respectively.

## 4. Discussion

The study aimed to present a non-invasive method that allows early detection of MetS without the need for invasive techniques. In this sense, we have proposed two algorithms and a discriminant model based on non-invasive variables (BMI, WHtR, and BP).

The prevalence of MetS rose to 26.7%, with a higher proportion in the female population, something observed in other works [27,28]. Although this prevalence is different from that shown by other authors, it depends mainly on the diagnostic criteria used, and the characteristics of the population studied [28,29]. Eyzaguirre et al. found a prevalence of 22.7% in the study of obese Chilean children, similar to that observed in our results [12]. Bustos et al. observed a proportion of 44% in another population with similar characteristics [13]. As demonstrated by our results, the literature has shown that overweight and obesity are the most important characteristics that contribute to the increase in the presence of MetS in children [30]. Therefore, we consider that the high prevalence of overweight and obesity in our sample (67%) has influenced the high presence of MetS (OR = 22.236). This trend has been observed by other authors [27,28,30,31].

This study has also revealed a strong association of all non-invasive variables (anthropometric and blood pressure) with MetS. Of these, BMI (AUC = 0.827) and WC (AUC = 0.808) presented the highest predictive capacity. Our work obtained a sensitivity of 86.4% for BMI (cut-off point of 23.5 kg/m^2^) and 83.1% for WC (cut-off point of 77.9 cm). Radetti et al. evidenced that the BMI, despite its limitations, showed better validity indexes in the prediction of MetS than other adiposity markers, emphasizing the ease of calculating BMI [32]. Other authors have indicated that this parameter (following different criteria) is a good predictor of the MetS components [33,34]. In the same vein, several investigations have highlighted the role of waist circumference and its ratio to height in the prediction of MetS [35,36]. However, various meta-analyses have shown that the cut-off points of these variables vary according to the reference population, making it difficult to compare our results’ predictive capacity with those obtained in other research [37,38].

These results are reinforced by those found in the different multivariate models described. Concerning the logistic regression models, adjusting for the variables mentioned above (WC, quantitative; HBP; qualitative; BMI; qualitative), we reached 66.1%, 90.7%, and 84.2% of sensitivity, specificity, and validity index, respectively. Additionally, the importance of BMI and altered blood pressure is perceived in the discriminant analysis. These variables, in its quantitative version, were capable of correctly discriminating against 76.9% of children. The inclusion of blood pressure in non-invasive diagnostic models is crucial because, as other authors have pointed out, HBP values are frequent in obese children suffering from MetS [12,14,39].

Moreover, data show the critical role of BMI, WC, WHtR, and BP in the early non-invasive diagnosis of MetS. However, the application of multivariate models in some clinical settings (schools, fieldwork, etc.) could be complicated by the need for mathematical calculations.

For this reason, two algorithms were performed to facilitate the clinical decision in the diagnosis. The first one was based on three variables (BP, BMI, and WHtR). In contrast, two variables (BMI and WHtR) were used to perform the second one. Wicklow et al., in a prospective study, showed that these variables are related to the appearance over time of cardiometabolic risk factors, which would justify their use in children, as is currently done in the adult population [15,16,18]. This fact is essential in our study population, given that, from the age of 9, the public education system does not maintain health control within healthy children program. Although in other populations, WC and WHtR have been proposed as an alternative to BMI in MetS discrimination, our results have shown that their combined use can increase their discriminatory capacity [40,41].

Regarding its applicability, the first of them, with high sensitivity (86.4%), could facilitate the screening of children suffering from MetS, even in clinical settings where blood pressure monitors are not available. On the other hand, the second algorithm presented a higher validity index (80.5%), allowing the proper classification of a higher number of cases correctly. This tree is recommended in situations where blood pressure measures are available.

In summary, from our point of view, this work puts at the disposal of the scientific community the first tools and the development of a methodology that would help in the early detection of MetS in children, something demanded by several authors [42,43]. Specifically, Ahadi et al. and Bianchini et al. encourage the routine use of non-invasive variables to screen metabolic risk factors and MetS in childhood [44,45]. However, given the phenotypic variability between different geographical locations, adaptations should be made according to the reference populations (hence the importance of this methodological approach). The inclusion of other variables associated with cardio-metabolic risks, such as neck circumference, could be considered [46]. The importance of early screening lies in achieving a good state of health and avoiding the possible alterations that develop in the following stages of life [47]. As we commented previously, from 9 onwards, the girls and boys do not continue the health control. For this reason, this proposal could have a high impact since it is based on quick and simple techniques that could be applied in schools by nurses, nutritionists, or even adequately trained teachers.

The proposed screening methods could facilitate early detection in the school setting, referral to primary care, and early intervention.

### Limitations

The sample presents a high prevalence of overweight and obesity, which raises the prevalence of MetS above that shown in the general child population. Besides, the study only included a population from South America. Given the heterogeneity of criteria for the diagnosis and the scarce consensus around them, the modified Cook’s criteria were used to facilitate comparison with other works carried out in similar populations. The CHAID methodology used to elaborate decision trees recommends large sample sizes to optimize statistical significance. In the sample used in this study (*n* = 221), the criteria for forming the parent and child nodes were low (50 and 30, respectively). Based on this, future work should consider larger samples with better representation of nutritional status and other ethnicities to contrast the proposed method more reliably. It is important to highlight as a strength that, to our knowledge, these models are the first tools for detecting MetS presented for Chilean children.

## 5. Conclusions

The prevalence of MetS is high in this child population, especially among those children who are overweight and obese. Two models have been found based on non-invasive variables that can be easily measured in any context (school, primary care, etc.). They present a good diagnostic precision and the potential to reduce the blood extractions involved (they would only be indicated for diagnostic confirmations). Therefore, they represent convenient and cost-efficient methods.

## Figures and Tables

**Table 1:** Characteristics of the sample by MetS and (crude and adjusted) logistic regression.

Raw Regression (No Adjusted)							Adjusted Regression		
Variables	Total (*n* = 221)	Presence (*n* = 59)	Absence (*n* = 162)	OR	95% CI	*p*	OR	95% CI	*p*
	Mean or *n* (SD or %)	Mean or *n* (SD or %)	Mean or *n* (SD or %)						
**Sex**									
**Women**	111 (51.2)	38 (64.4)	73 (45.1)	2.206	1.191–4.086	<0.05	3.488	1.586–7.674	<0.01
**Men**	110 (49.8)	21 (35.6)	89 (54.9)	1	1		1	1	
**Age (years)**	9.1 (1.3)	9.3 (1.2)	9 (1.3)	1.217	0.949–1.559	NS			
**Weight (Kg)**	41.2 (10.8)	49.2 (9)	38.3 (9.9)	1.124	1.081–1.168	<0.001			
**Height (cm)**	135.2 (9)	138.2 (8.6)	134.1 (8.9)	1.057	1.019–1.097	<0.01			
**WC (cm)**	74.6 (11.5)	83.6 (10.8)	71.3 (10.8)	1.131	1.086–1.177	<0.001			
**WHtR > 0.55**	128 (57.9)	55 (93.2)	73 (45.1)	16.764	5.801–48.441	<0.001			
**BMI (Kg/m^2^)**	22.2 (4.2)	25.6 (3.1)	21 (3.8)	1.435	1.276–1.613	<0.001	1.380	1.219–1.563	<0.001
**Overweight/Obesity**	148 (67)	57 (96.6)	91 (56.2)	22.236	5.249–94.206	<0.001			
**BF (%)**	30.1 (9.4)	36.6 (6)	27.7 (9.4)	1.139	1.087–1.193	<0.001			
**FM (Kg)**	13.4 (6.7)	18.3 (5.7)	11.5 (6.2)	1.194	1.124–1.268	<0.001			
**FFM (Kg)**	28.4 (5.1)	31.1 (5.1)	27.4 (4.7)	1.173	1.093–1.258	<0.001			
**SBP (mmHg)**	101.5 (12)	108.7 (12)	98.9 (10.9)	1.086	1.051–1.121	<0.001	1.059	1.022–1.098	<0.001
**DBP (mmHg)**	66 (10.8)	71.1 (10.7)	64.1 (10.2)	1.073	1.037–1.109	<0.001			
**Glucose (mg/dL)**	88.2 (8.5)	88.6 (10)	88.1 (8)	1.006	0.972–1.042	NS			
**CholT (mg/dL)**	181.7 (33.1)	188.2 (36.4)	179.3 (31.6)	1.008	0.999–1.017	NS			
**Chol-HDL (mg/dL)**	50.5 (11.6)	40.4 (10.2)	54.2 (9.8)	0.859	0.821–0.899	<0.001			
**Chol-LDL (mg/dL)**	108.2 (28.8)	113.3 (32.9)	106.3 (27.1)	1.008	0.998–1.019	NS			
**Triglycerides (mg/dL)**	122.9 (73.8)	186 (93.1)	99.9 (48.1)	1.020	1.014–1.026	<0.001			

MetS: Metabolic Syndrome; SD: Standard Deviation; OR: Odds Ratio; WC: Waist circunference; WHtR: Waist to height ratio; BMI: Body Mass Index; BF: Body fat; FM: Fat Mass; FFM: Fat Free Mass; SBP/DBP: Systolic/Diastolic Blood Pressure; CholT: Total Cholesterol.

**Table 2:** Multivariate analysis and adjusted logistic regression for non-invasive categorical (dichotomous) variables.

Model 1				
Variable	Coefficient	OR	95% CI	*p*
**Sex (Woman)**	1.114	3.047	1.392–6.669	<0.01
**WHtR ≥ 0.55**	1.714	5.551	1.996–15.442	<0.01
**BP ≥ 104.5/69**	1.448	4.253	1.743–10.375	<0.01
**BMI ≥ 23.5**	1.411	4.544	1.120–14.999	<0.05
**Model 2**				
**Sex**	1.263	3.535	1.511–8.273	<0.01
**WC**	0.076	1.079	1.020–1.142	<0.01
HBP *	1.941	6.964	3.012–16.104	<0.001
**BMI ≥ 23.5**	1.235	3.443	1.070–11.081	<0.05
**Sensitivity, Specificity and Validity Index for the Predictive MetS Model**				
	**Model 1**		**Model 2**	
**Sensitivity**	45.8%		66.1%	
**Specificity**	95.1%		90.7%	
**Validity index**	81.9%		84.2%	
**R^2^ Nagelkerke**	0.455		0.511	
**R^2^ Cox-Snell**	0.313		0.350	
**Hosmer-Lemeshow (*p*)**	0.734		0.417	

WHtR: Waist to height ratio; BP: Blood Pressure; BMI: Body Mass Index; WC: Waist circumference; BF: Body fat; Metabolic Syndrome; OR: Odds Ratio; CI: Confidence interval; * High Blood Pressure according to age, sex and height; Models adjusted by sex and age.

**Table 3:** Discriminant Analysis.

Variable	MetS [No] Coefficient	MetS [Yes] Coefficient	*p*
**BMI**	0.960	1.251	<0.001
**SBP**	0.674	0.721	<0.001
**Constant**	−44.106	−55.778	-
**Sensitivity, Specificity and Validity Index for the Predictive MetS Model**			
**Sensitivity**	81.4%		
**Specificity**	75.3%		
**Validity index**	76.9%		

BMI: Body Mass Index; WC: Waist circumference; SBP: Systolic Blood Pressure; MetS: Metabolic Syndrome.
